# Screening and Bioinformatic Analysis of Drought-Adaptive-Response Genes in Wild *Cerasus humilis*

**DOI:** 10.3390/ijms27188186

**Published:** 2026-09-15

**Authors:** Xu Wang, Wenzhe Feng, Li Fan, Yuedong Zhang, Xiaoyan Li, Haiqing Tian

**Affiliations:** 1 College of Mechanical and Electrical Engineering, Inner Mongolia Agricultural University, Hohhot 010018, China; wx_xhl@163.com; 2 College of Horticultural and Plant Protection, Inner Mongolia Agricultural University, Hohhot 010018, China

**Keywords:** wild Prunus cerasifera, cDNA library, yeast high-throughput sequencing, drought adaptation-associated gene

## Abstract

This study aimed to screen genes associated with drought adaptation in *Cerasus humilis* and analyze their sequence characteristics and evolutionary relationships. The results could provide a theoretical basis for exploring the molecular basis underlying drought adaptation of *Cerasus humilis*. Two-year-old wild *Cerasus humilis* plants were cultivated in a greenhouse, and irrigation was completely withheld for 20 consecutive days. Leaves were sampled and a cDNA library of wild *Cerasus humilis* was constructed. By utilizing high-throughput screening in yeast combined with mannitol-induced simulated drought stress, genes associated with drought adaptation were isolated and identified. Additionally, core candidate genes were cloned and subjected to systematic bioinformatic analysis. The results showed that: (1) the drought adaptation-associated genes *ChU40rps27a* and *ChTp258* were obtained through high-throughput screening of yeast and mannitol-simulated drought resistance test; (2) gene cloning revealed that the open reading frame of *ChU40rps27a* is 471 bp long, encoding 157 amino acids, and the open reading frame of *ChTp258* is 231 bp long, encoding 77 amino acids; (3) multiple sequence alignment of the genes indicated that *ChU40rps27a* shares 88.5% identity with apple 40S (XM_050288755), and *ChTp258* shares 94.4% identity with peach (XM_020567676) in terms of nucleotide sequence; (4) the phylogenetic tree analysis revealed that *ChU40rps27a* is closely related to *Citrus sinensis* *U40rps27a* (XM_006487968); and *ChTp258* is closely related to peach (*Prunus persica*, XM_020567676), apricot (*Prunus dulcis*, XM_034370534), plum (*Prunus mume*, XM_008244122), and sweet cherry (*Prunus avium*, XM_021951344) in the Rosaceae family, clustering in the same clade. This study identified two candidate genes involved in long-term drought adaptation rather than short-term stress responses. These findings clarify their sequence characteristics, sequence conservation and evolutionary status, providing important gene resources and theoretical support for further exploring drought adaptation related molecular processes in *Cerasus humilis* and developing drought-adapted molecular breeding.

## 1. Introduction

Chinese dwarf cherry (*Cerasus humilis*) is a deciduous shrub belonging to the genus Prunus within the family Rosaceae. It is a unique and economically valuable forest tree species in China [1], distributed across 13 provinces, municipalities, and autonomous regions including Shandong, Hebei, and Inner Mongolia. Possessing remarkable characteristics such as strong cold resistance, drought tolerance, and adaptability to poor soils [2], it is an ideal species for ecological restoration and economic cultivation in arid and semi-arid regions. Its fruits are rich in calcium [3], anthocyanins [4], flavonoids, vitamins, and various amino acids [5], offering high nutritional value and a distinctive flavor. With both ecological and economic benefits, it holds significant application prospects in the development of specialty forest fruit industries [6].

Drought stress is a major abiotic adversity that severely affects the growth and development of Chinese dwarf cherry, limiting its cultivation distribution, yield, and quality [7]. Under prolonged drought conditions, plants often exhibit stress responses such as inhibited growth [8], decreased photosynthetic rate, intensified membrane lipid peroxidation [2], and physiological metabolic disorders. Extreme drought can lead to growth decline or even plant death [9]. As an important ecological and economic dual-purpose fruit tree in northern China’s arid and semi-arid regions, elucidating its intrinsic molecular basis for drought adaptation and identifying key genes associated with drought adaptation are of great theoretical and practical value for enhancing its drought adaptability and ensuring stable, high-quality production. Previous studies in species such as maize [10], apple [11], and Populus trichocarpa [12] have demonstrated that transcription factors like MYB, WRKY, NAC, as well as functional genes such as ribosomal proteins and transmembrane proteins, modulate plant drought adaptive capacity through changes in their gene expression, providing important references for research on drought-resistant genes in Chinese dwarf cherry.

Currently, research on drought resistance in Chinese dwarf cherry primarily focuses on germplasm evaluation, hybrid breeding, and physiological-biochemical index measurement [13]. Studies exploring drought adaptation-associated functional genes, cell type specific molecular responses and regulatory networks in *Cerasus humilis* and related Cerasus species remain scarce. Most existing works only report physiological phenotypes, while candidate gene validation (such as gene knockout or gene silencing) is still limited, which restricts our understanding of the molecular basis of drought adaptation for this species. Studies on the molecular mechanisms of drought response, mining of key functional genes, and analysis of regulatory networks remain relatively limited [14], which hinders the advancement of molecular breeding for drought resistance and research on stress tolerance mechanisms. Therefore, this study utilized wild Chinese dwarf cherry as material to construct a cDNA library under drought stress. Employing yeast high-throughput screening combined with a mannitol-simulated drought system, genes associated with drought adaptation were isolated and identified. Candidate genes were cloned and subjected to bioinformatics analysis to predict their protein physicochemical properties, conserved domains, transmembrane characteristics, phosphorylation sites, and phylogenetic relationships. The aim is to identify key candidate genes involved in drought adaptation in Chinese dwarf cherry, analyze their sequence features and evolutionary patterns, and provide gene resources and a theoretical foundation for clarifying molecular processes of drought adaptation and breeding new drought-resistant, high-yield varieties.

## 2. Results

### 2.1. Construction of Cerasus humilis cDNA Library

#### 2.1.1. RNA Quality Detection

**Figure 1:**
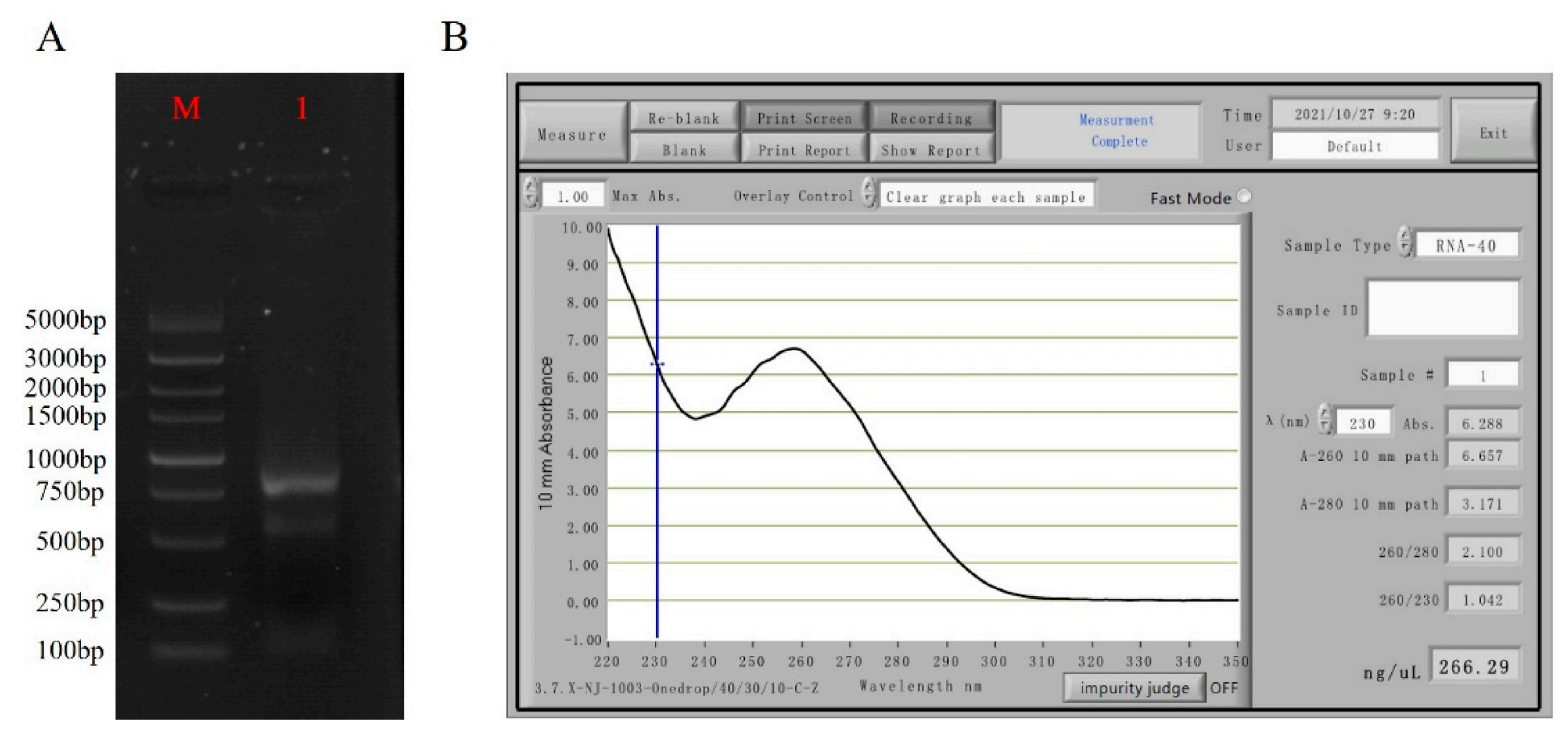
RNA extraction. Note: (**A**) RNA electrophoresis detection result, M: Marker, 1: Total RNA of *Cerasus humilis* sample. (**B**) RNA determination of *Cerasus humilis* sample by spectrophotometer.

Agarose gel electrophoresis (Figure 1A) showed that the extracted total RNA of *Cerasus humilis* presented distinct and bright 28S and 18S rRNA bands, while the 5S rRNA band was faint, indicating good RNA integrity without obvious degradation. Ultraviolet spectrophotometry detection (Figure 1B) revealed that the OD_260_/OD_280_ ratio was 2.10, the OD_260_/OD_230_ ratio was 1.04, and the RNA concentration was 266.29 ng/μL. These results demonstrated that the RNA possessed high purity and was free of contamination by protein, polysaccharides and other impurities, which met the requirements for subsequent cDNA library construction.

#### 2.1.2. Double-Stranded cDNA Synthesis

**Figure 2:**
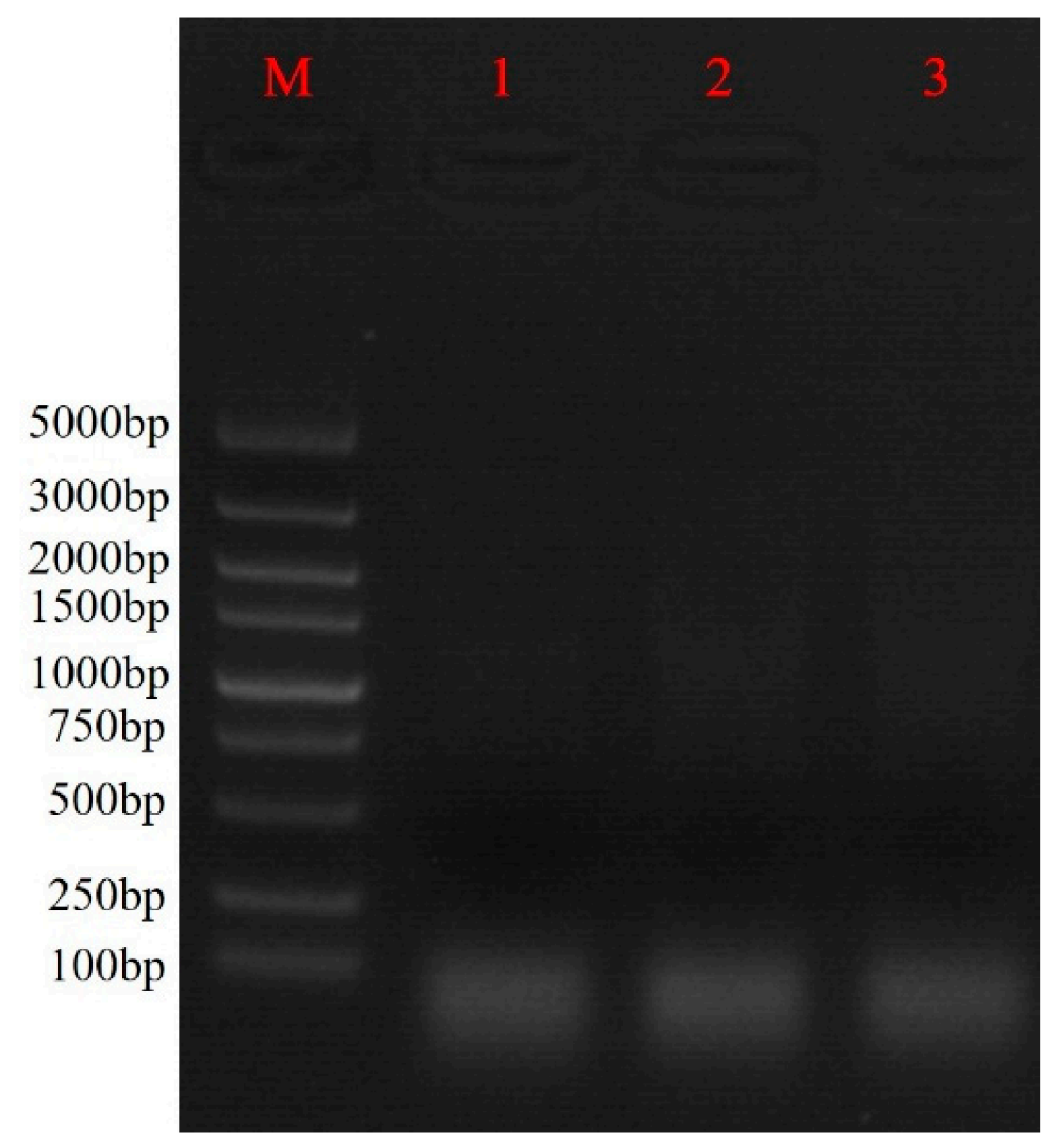
ds cDNA synthesis. Note: The quality of ds cDNA was assessed by agarose gel electrophoresis. M: Marker; 1: ds cDNA amplified by P1-F/P4-R; 2: ds cDNA amplified by P2-F/P4-R; 3: ds cDNA amplified by P3-F/P4-R.

The double-stranded cDNA products were detected by agarose gel electrophoresis (Figure 2), showing a clear diffuse distribution with a wide band coverage range. This indicated that the synthesized ds cDNA fragments had a uniform size distribution and high quality, which could meet the requirements of subsequent normalization and library construction.

#### 2.1.3. ds cDNA Normalization and Small Fragment Removal

**Figure 3:**
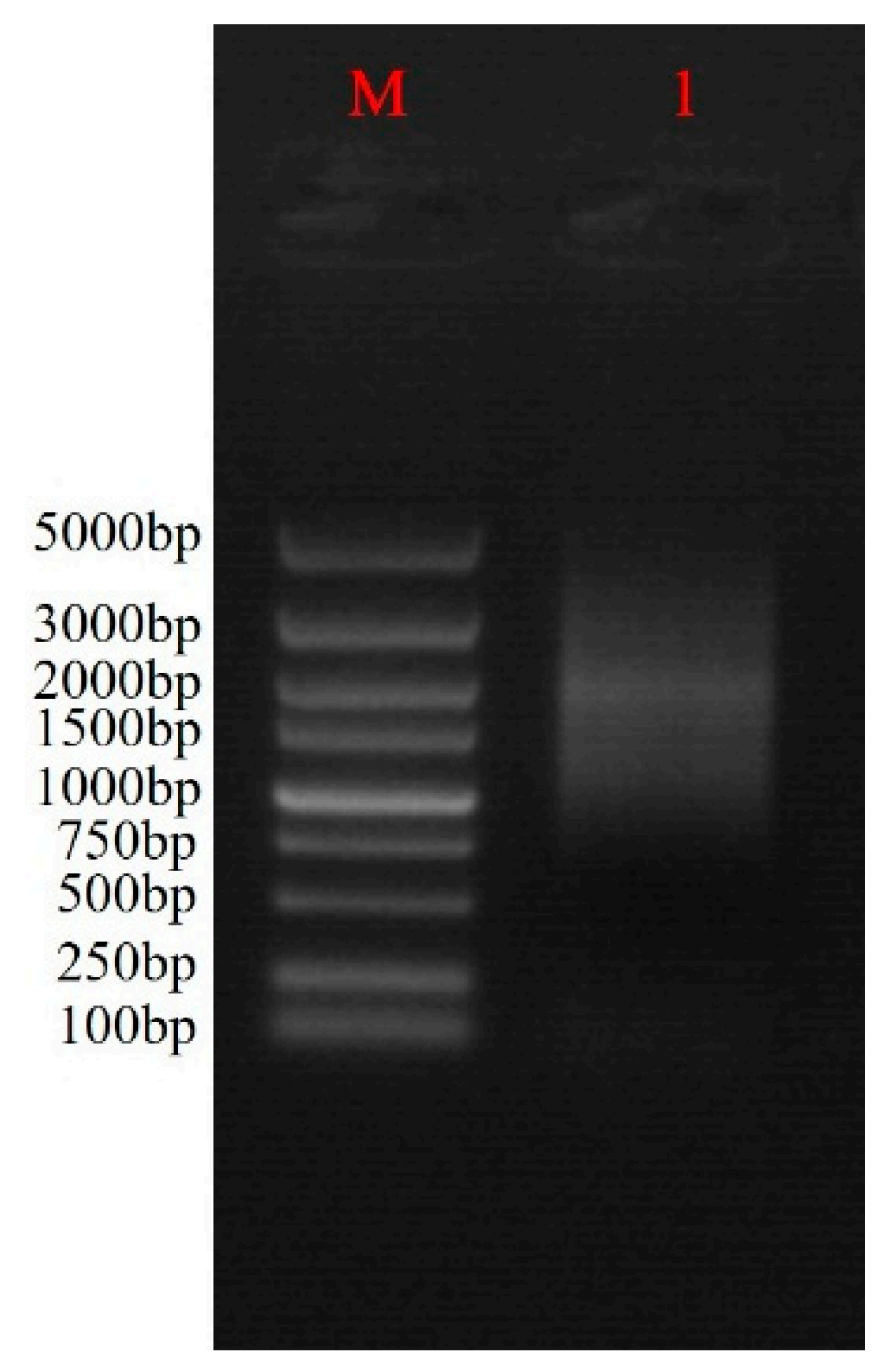
Homogenization and removal of small fragments detected by agarose gel electrophoresis. Note: Agarose gel electrophoresis was used to evaluate the effects of normalization and small fragment removal of ds cDNA. M: Marker; 1: Purified product of the mixed three types of ds cDNA.

The double-stranded cDNA after normalization and small fragment removal was examined by agarose gel electrophoresis (Figure 3). The products displayed a homogeneous diffuse smear without obvious bright band enrichment, indicating an effective normalization performance. Meanwhile, bands below 500 bp were nearly undetectable, suggesting that small fragments were efficiently removed, and the obtained cDNA was qualified for subsequent library construction.

#### 2.1.4. Clone Counting

To determine the library capacity, 10 μL of electrotransformed bacterial solution was diluted 10^4^-fold, and 100 μL of the dilution was spread onto LB plates containing ampicillin (Amp). The plates were incubated overnight at 37 °C in an inverted position. Colony counting yielded a total of 452 single colonies on the plate. According to calculation, the library titer was 4.52 × 10^7^ cfu/mL, with a total library capacity of 9.04 × 10^7^ cfu. The results indicated that the library capacity met the requirements for subsequent screening.

#### 2.1.5. Library Quality Evaluation

**Figure 4:**
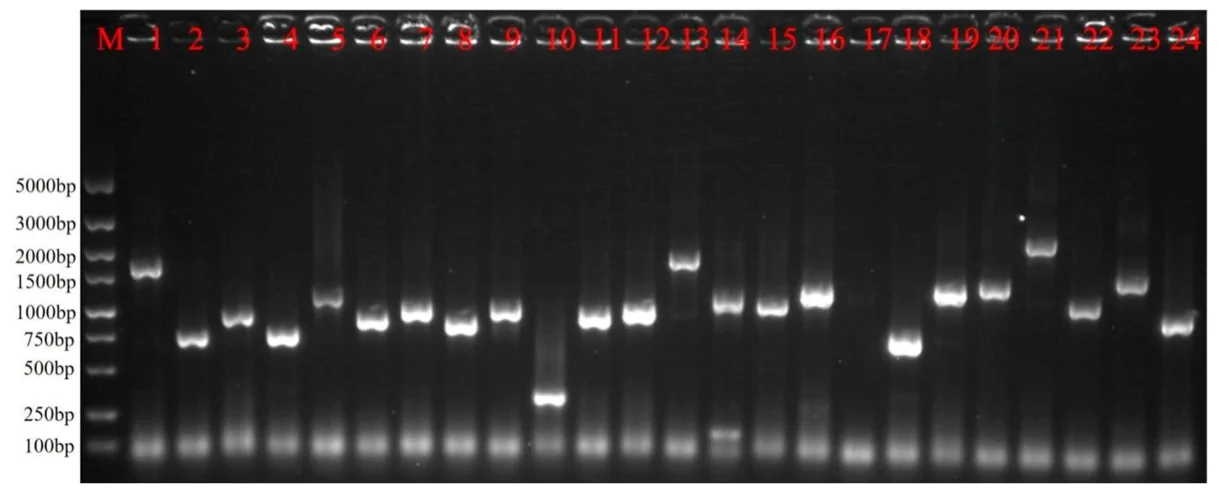
Library quality evaluation. Note: Agarose gel electrophoresis of Escherichia coli colony PCR products. M: Marker; 1–24: PCR products of 24 randomly selected single colonies.

Twenty-four single colonies were randomly selected for colony PCR using the primer pair T7-F/AD-R. PCR products were analyzed by agarose gel electrophoresis (Figure 4). Except for Lane 17 with no target band, clear amplified bands were obtained from the remaining 23 clones. The inserted fragment size was mainly distributed around 1000 bp. The average insert size of the library was approximately 1000 bp, with a recombination rate of 96%, indicating that the constructed cDNA library met the quality criteria for a high-quality cDNA library.

### 2.2. High-Throughput Screening of Drought-Tolerant Genes in Yeast

#### 2.2.1. Screening of Optimal Mannitol Concentration

**Figure 5:**
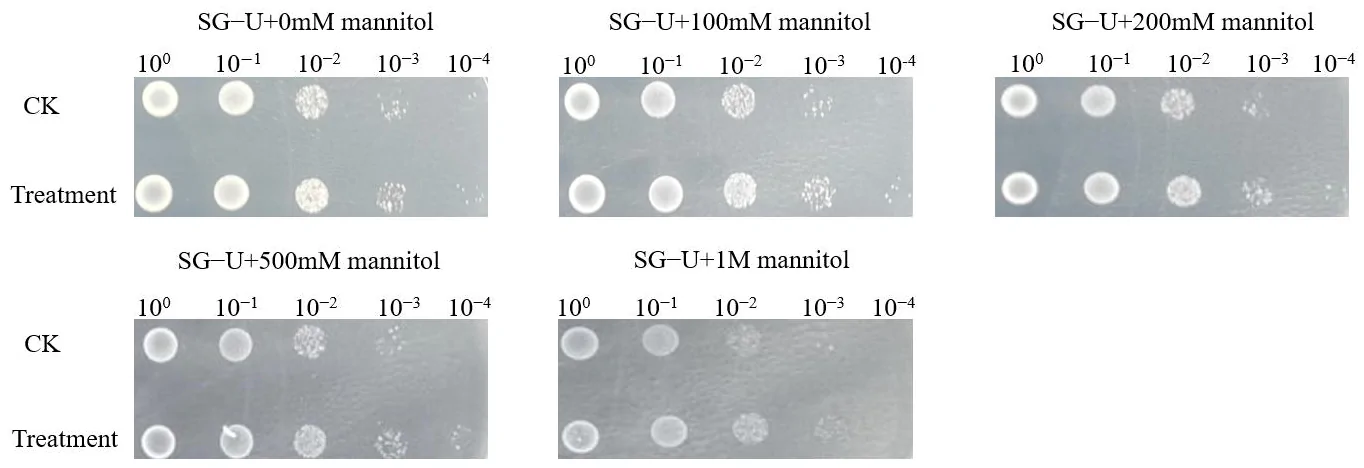
Condition filtering results. Note: The control group here refers to yeast transformed with empty pYES2 vector without Cerasus humilis cDNA insert.

Under gradient mannitol stress, the spotting results of yeast suspensions with different dilution gradients are shown in Figure 5. In the control group, no obvious colony growth was observed at the dilution of 10^−4^ under 100 mM mannitol. In contrast, the experimental group grew normally at the same mannitol concentration and dilution gradient and exhibited a prominent drought tolerance advantage. These results indicated that 100 mM mannitol was the suitable mannitol level for screening yeast clones with enhanced survival under osmotic stress.

#### 2.2.2. BLAST Analysis of Sequencing Results

DNA sequencing was performed on positive yeast clones, and the obtained sequences were aligned against the GenBank database to achieve accurate gene annotation. A total of 96 positive yeast clones were obtained and subjected to PCR amplification. Subsequently, sequence assembly and alignment were conducted using SeqMan( version Pro 12.3) and BLAST (version 2.14.1) programs. After removing empty vector sequences and redundant repeats, 44 unique sequences were finally identified. The 44 annotated sequences are as follows: Probable aspartic proteinase GIP2; COP9 signalosome complex subunit 7-like, transcript variant X3; Metallothionein-like protein type 2; Cultivar NCNSP0306 chromosome 14; 60S ribosomal protein L19-1, transcript variant X2; Probable phytol kinase 1; Thioredoxin H2; E3 ubiquitin-protein ligase RNF149; Cyclophilin; Cultivar NCNSP0306 chromosome 8; Protein RNA-directed DNA methylation 3; Serine carboxypeptidase-like 27; Protein LSM12 homolog; Serine/threonine-protein kinase SAPK2; Ubiquitin-40S ribosomal protein S27a; Crocetin glucosyltransferase; Glycine-rich RNA-binding protein 4; 39S ribosomal protein L41-A; Calmodulin-7; Phosphatidyl-N-methylethanolamine N-methyltransferase; Ribulose bisphosphate carboxylase small chain SSU1A1 (two sequences); Soluble inorganic pyrophosphatase (two sequences); 60S ribosomal protein L19-1; Metallothionein-like type 1 protein (two sequences); Monothiol glutaredoxin-S7; Thioredoxin H2 (two sequences); Cultivar NCNSP0306 chromosome 13; Tubby-like F-box protein 5; CAAX prenyl protease 2; Calcineurin B-like protein 7; Metallothionein-like protein type 2; Histone acetyltransferase of the MYST family 1; Transmembrane protein 258; ACT domain-containing protein ACR9; Cultivar NCNSP0323 chromosome 10; Universal stress protein PHOS32; F1-ATPase epsilon-subunit; Carotenoid 9,10(9′,10′)-cleavage dioxygenase 1; Uncharacterized LOC116025535, transcript variant X2; and Protein BUNDLE SHEATH DEFECTIVE 2.

#### 2.2.3. Reversion Verification

**Figure 6:**
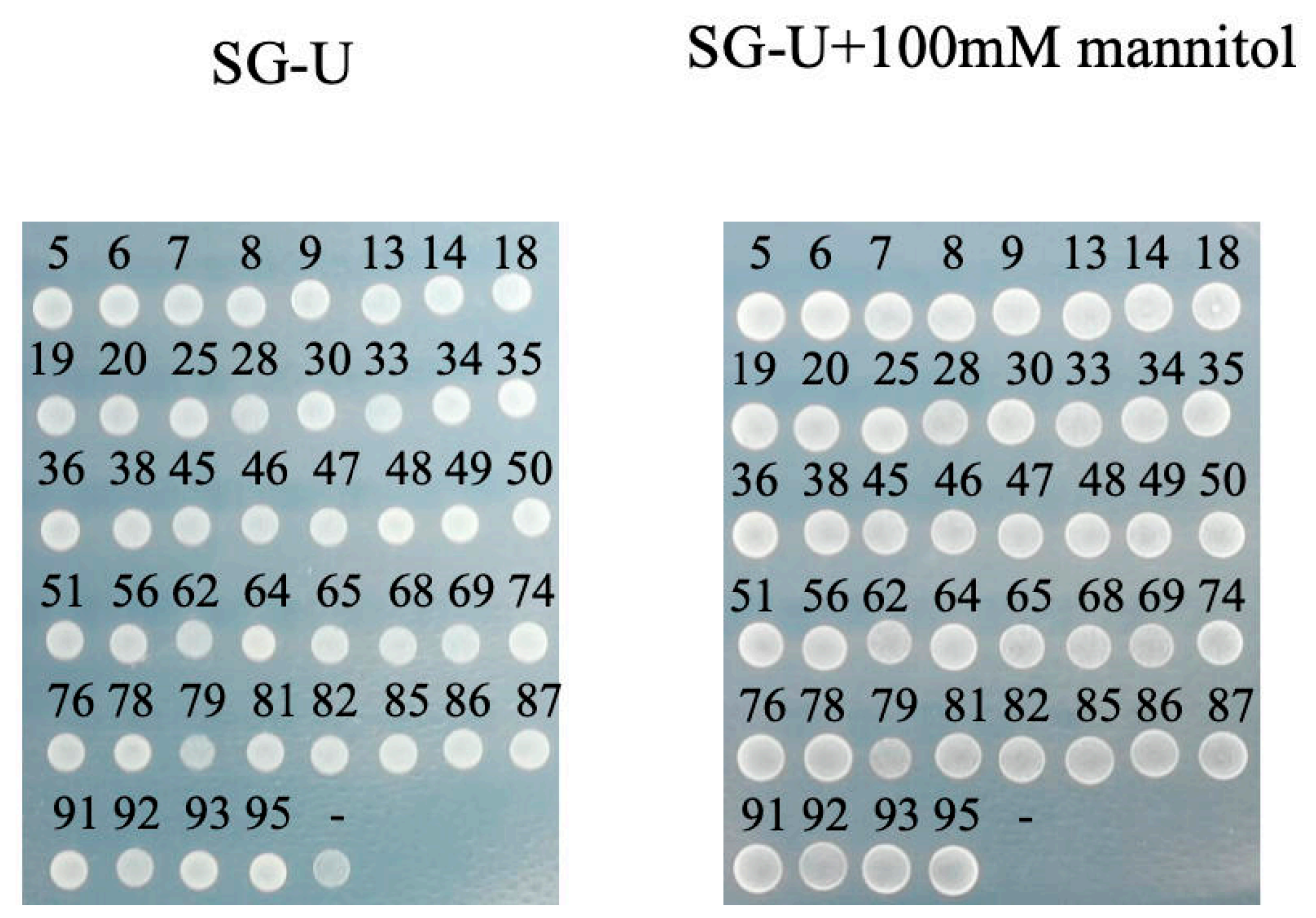
Verification result of rotation.

As shown in Figure 6, distinct growth differences were observed between the negative control strain and the positive clones in the reversion verification. Specifically, the negative control strain exhibited growth only on the basic SG-U medium and was completely unable to grow on the stress medium supplemented with 100 mM mannitol. In sharp contrast, all 44 positive clones screened out grew vigorously and normally on both the basic SG-U medium and the mannitol stress medium. These results clearly demonstrated that the positive clones possessed significant mannitol stress tolerance, thereby effectively verifying the reliability and accuracy of the primary screening results.

### 2.3. Cloning of ChU40rps27a and ChTp258 Genes from Cerasus humilis

**Figure 7:**
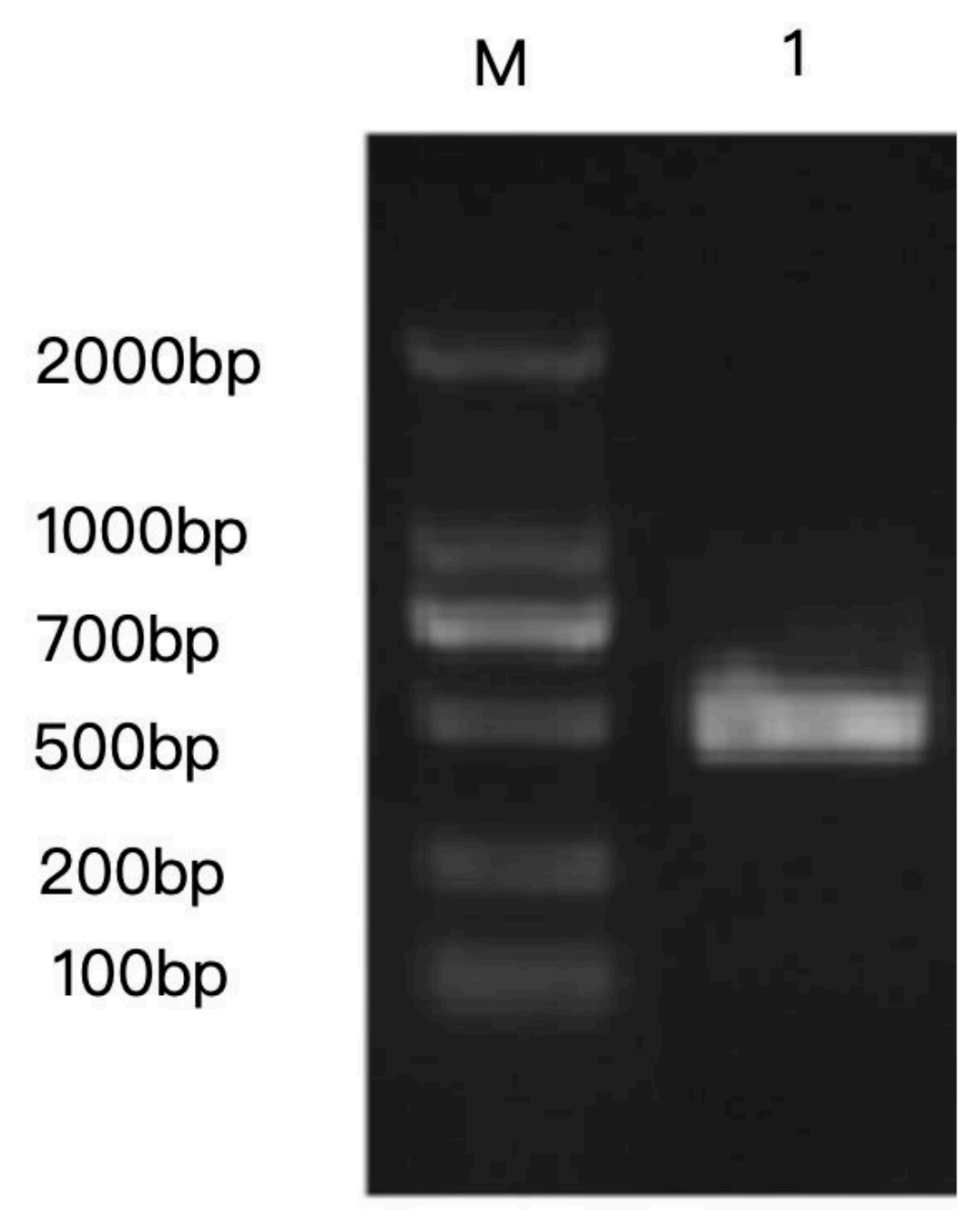
PCR amplification of the full-length ORF of ChU40rps27a. Note: The amplified products were detected by 1% agarose gel electrophoresis. M: DL2000 DNA Marker; 1: ORF fragment of the ChU40rps27a gene amplified using *Cerasus humilis* leaf cDNA as the template.

**Figure 8:**
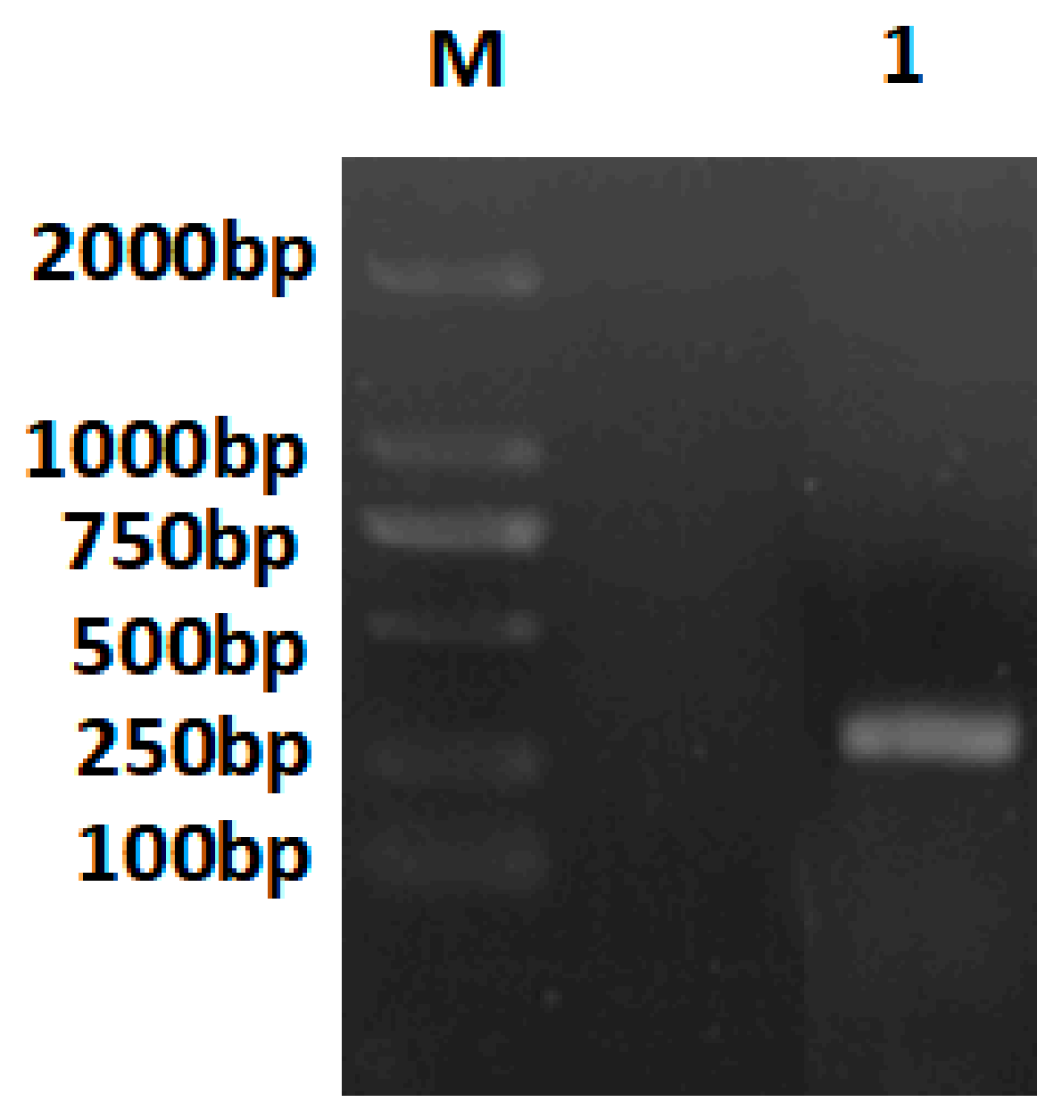
PCR amplification of the full-length ORF of ChTp258. Note: Amplified products were detected by 1% agarose gel electrophoresis. M: DL2000 DNA Marker; 1: ORF fragment of the ChTp258 gene amplified with *Cerasus humilis* leaf cDNA as the template.

Using cDNA derived from *Cerasus humilis* leaves as the template, the specific primer pairs ChU40rps27a-F/R and ChTp258-F/R were employed to amplify the full-length ORFs of the target genes. The amplified products were detected by 1% agarose gel electrophoresis (Figure 7 and Figure 8). The results showed clear and specific bands without non-specific amplification, and the fragment sizes were consistent with the expected lengths, indicating successful gene amplification. The target fragments were recovered by gel excision, ligated into the cloning vector, and sequenced to verify the accuracy of the obtained sequences.

### 2.4. Bioinformatics Analysis of ChU40rps27a and ChTp258 Genes from Cerasus humilis

#### 2.4.1. Prediction of Basic Properties of ChU40rps27a and ChTp258 Genes from *Cerasus humilis*

**Figure 9:**
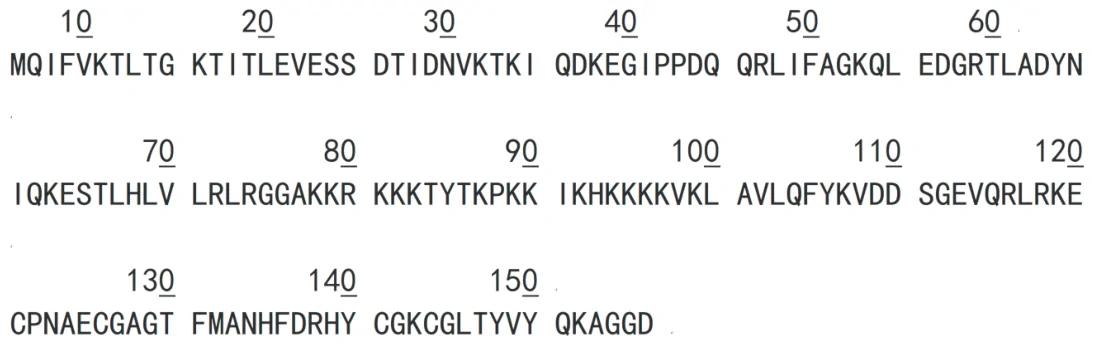
The nucleotide sequence and corresponding amino acid sequence of ChU40rps27 gene. Note: The start codon ATG and stop codon TAA are marked by underline and asterisk, respectively.

**Figure 10:**
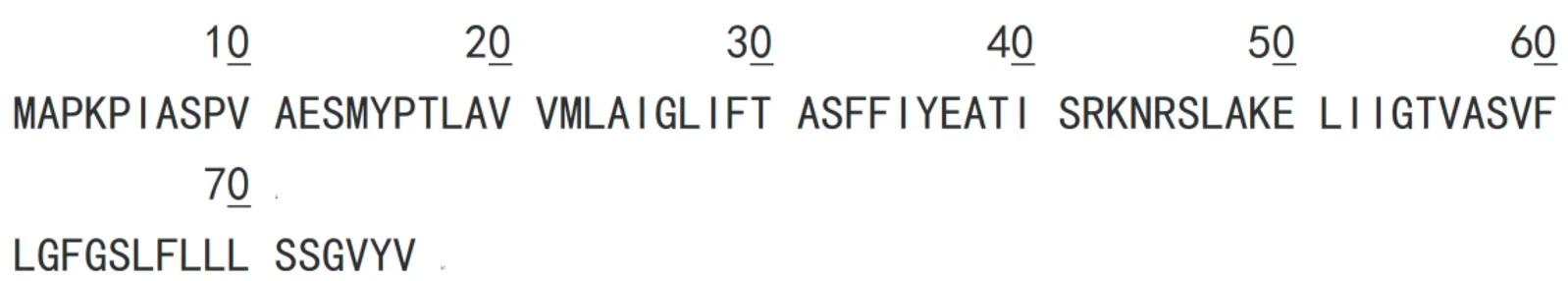
The nucleotide sequence and corresponding amino acid sequence of ChTp258 gene. Note: The start codon ATG and stop codon TAA are marked by underline and asterisk, respectively.

**Figure 11:**
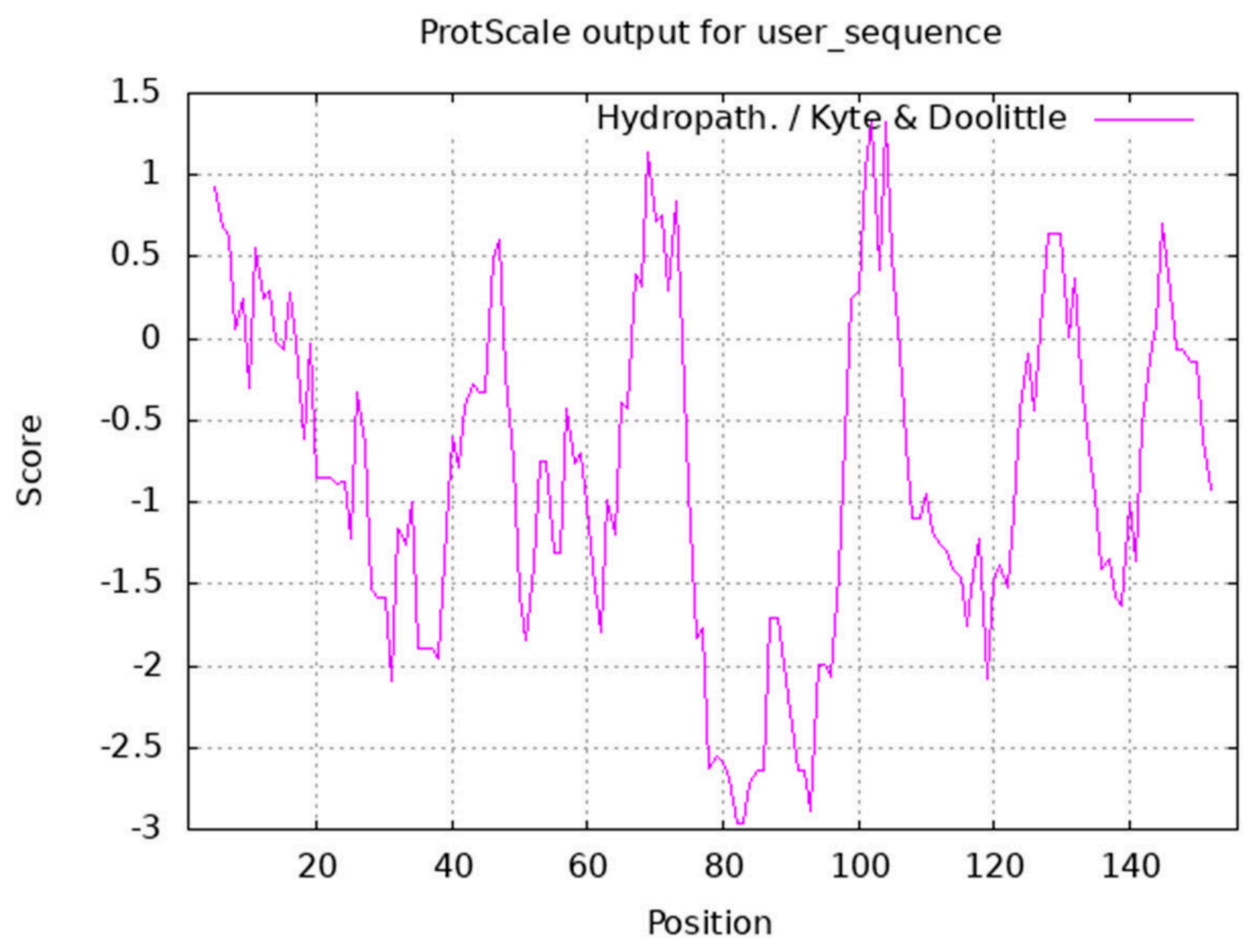
Analyses of hydrophily/hydrophpbicity of ChU40rps27a.

**Figure 12:**
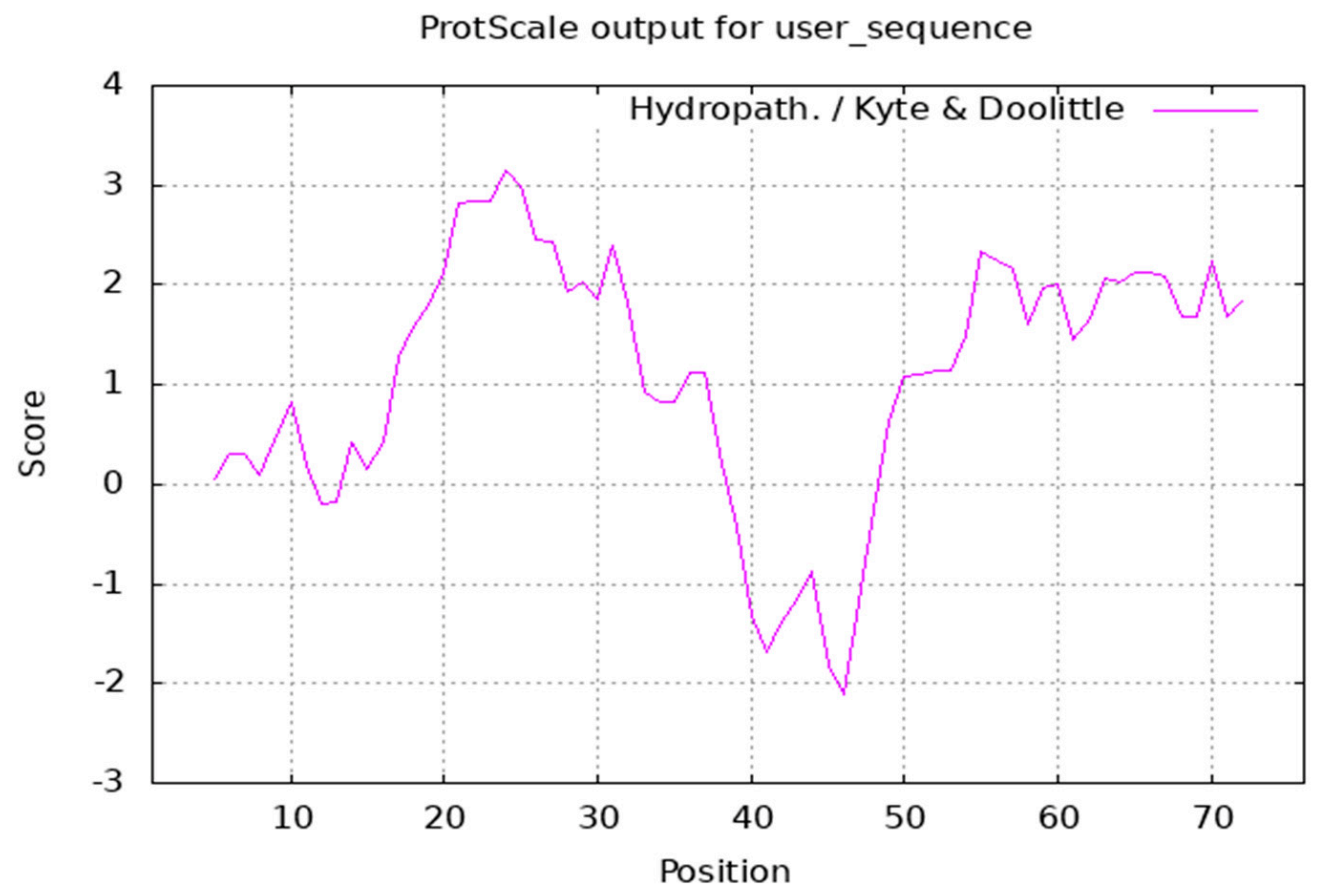
Hydrophilic hydrophobic profile analysis of ChTp258 protein.

Sequencing results (Figure 9 and Figure 10) showed that the open reading frame (ORF) of the ChU40rps27a gene was 471 bp in length, encoding 157 amino acids, while the ORF of the ChTp258 gene was 231 bp, encoding a total of 77 amino acids. Physicochemical property analysis (Figure 11 and Figure 12) revealed the following characteristics: the protein encoded by ChU40rps27a had a molecular formula of C_785_H_1288_N_226_O_228_S_6_, a molecular weight of 17.7 kDa, a theoretical isoelectric point (pI) of 9.72, an instability index of 34.00, and a grand average of hydropathicity (GRAVY) of 74.36. Among the amino acid composition, Lys (16.0%), Gly (8.3%), and Leu (8.3%) were the most abundant, and the number of positively charged residues (Arg + Lys, 33) was higher than that of negatively charged residues (Asp + Glu, 18). ProtScale hydrophobicity analysis indicated that the average hydrophilicity coefficient was −0.772, with the lowest hydrophobicity at amino acid positions 82–83 (−2.967) and the highest hydrophobicity at position 104 (1.322), suggesting that the ChU40rps27a protein was a hydrophilic protein overall.

For the protein encoded by ChTp258, its molecular formula was C_382_H_608_N_86_O_100_S_3_, with a molecular weight of 8.1 kDa, a theoretical pI of 9.30, an instability index of 36.37, and a GRAVY of 125.79. In terms of amino acid composition, Leu (13.2%), Ala (11.8%), and Ser (11.8%) were the most abundant, and the number of positively charged residues (Arg + Lys, 5) was higher than that of negatively charged residues (Asp + Glu, 3). ProtScale hydrophobicity analysis showed that the average hydrophilicity coefficient was 1.061, with the highest hydrophobicity at amino acid position 24 (3.144) and the lowest at position 46 (−2.111), indicating that the ChTp258 protein was a hydrophobic protein overall.

#### 2.4.2. Prediction and Analysis of Phosphorylation Sites and Functional Domains of ChU40rps27a and ChTp258 from *Cerasus humilis*

**Figure 13:**
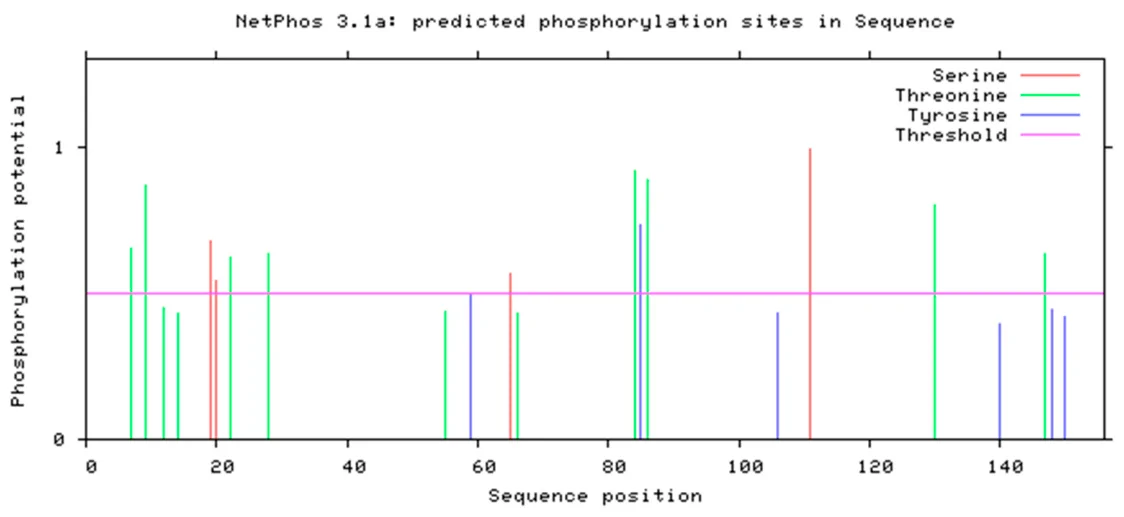
Prediction of phosphorylation sites of ChU40rps27a protein.

**Figure 14:**
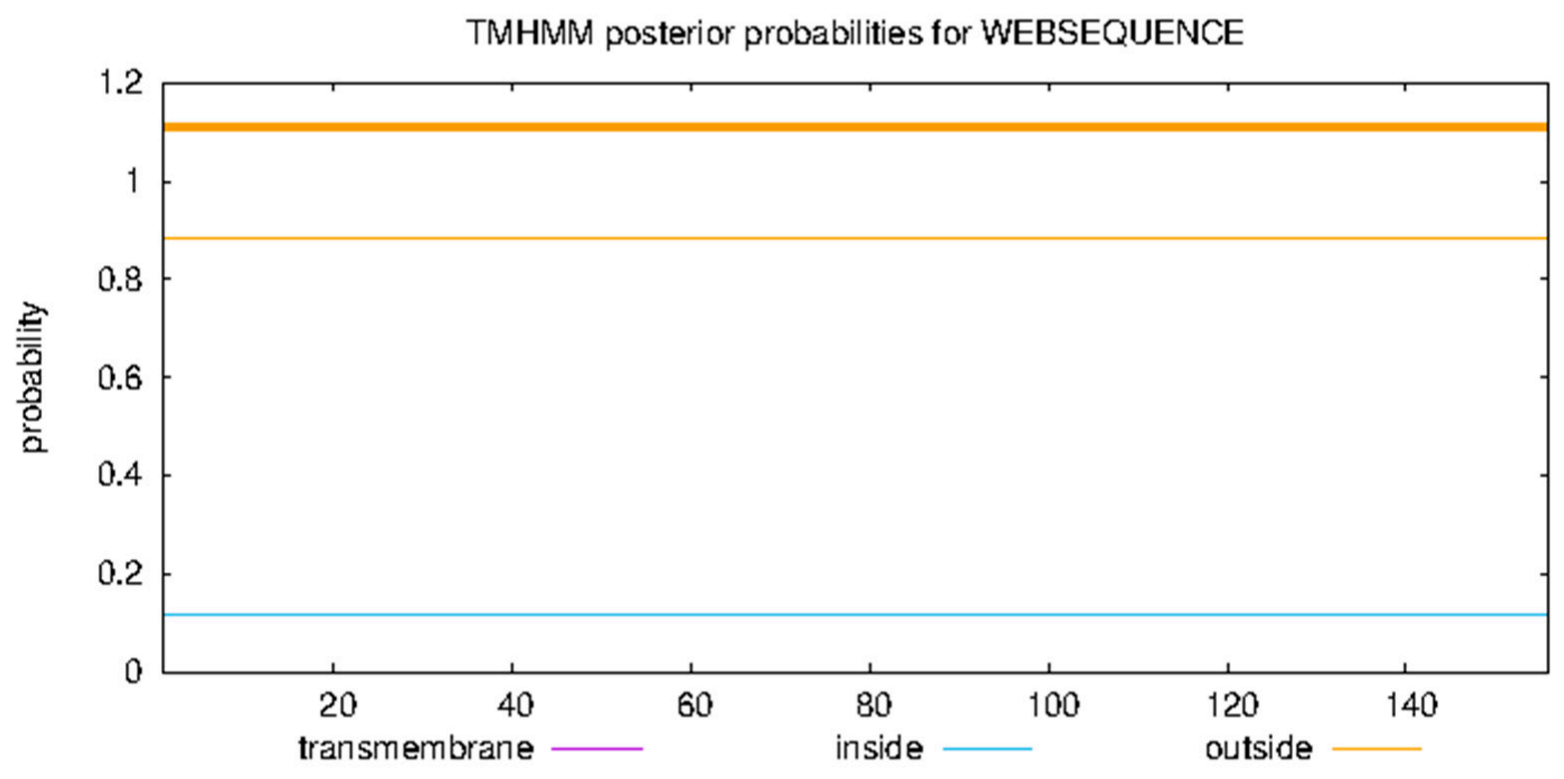
Prediction for transmembrane structures of ChU40rps27a.

**Figure 15:**
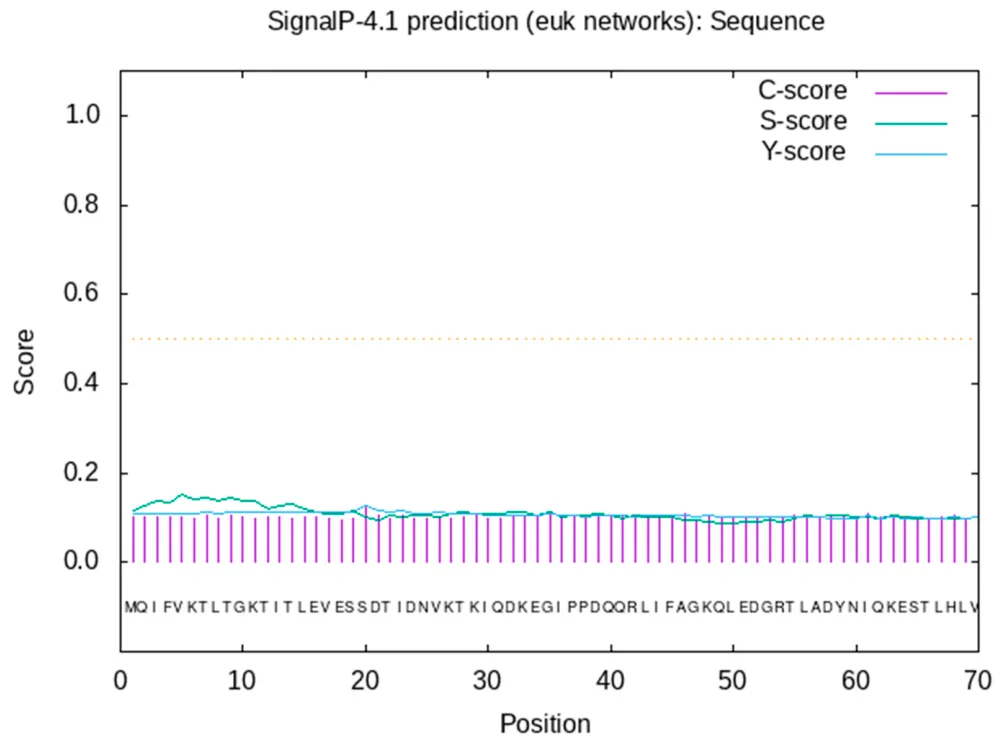
Prediction on signal peptide of ChU40rps27a protein.

**Figure 16:**
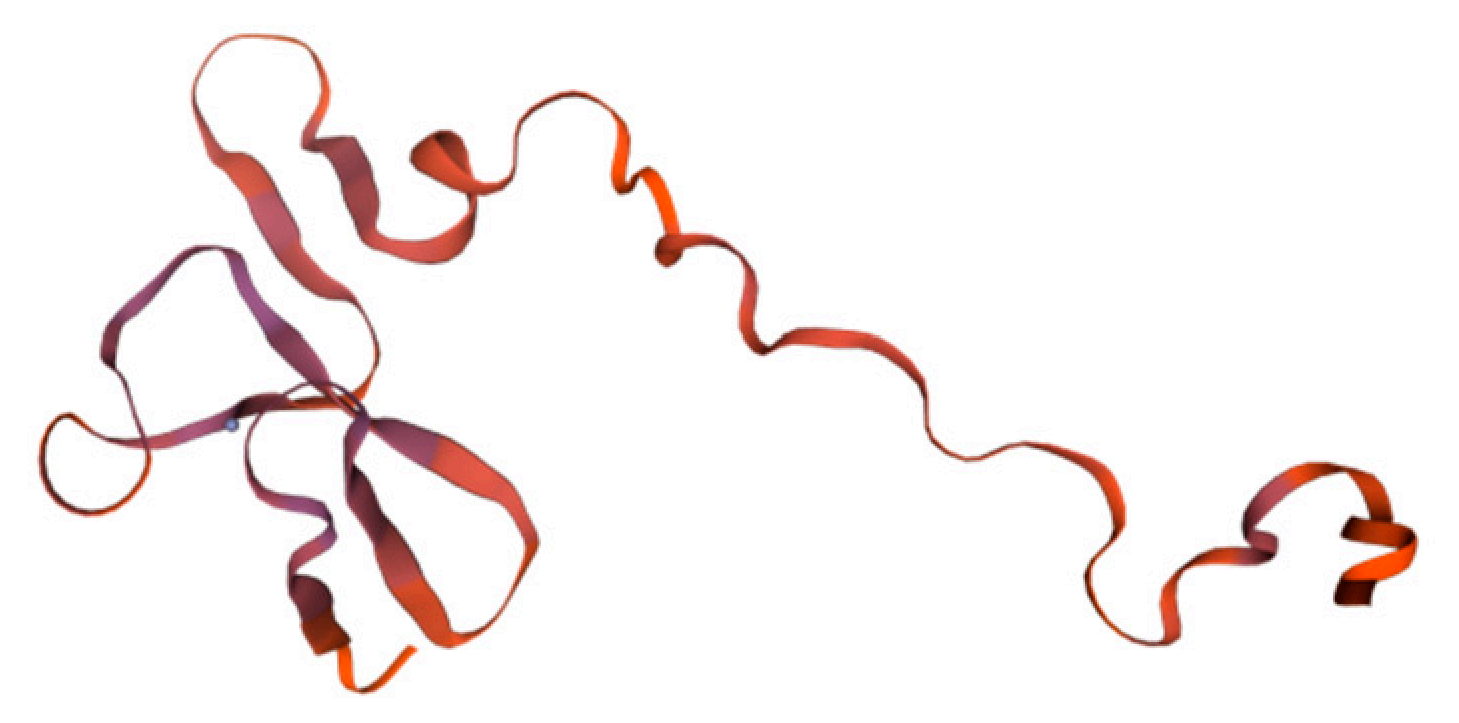
Three-dimensional structure diagram of ChU40rps27a.

**Figure 17:**
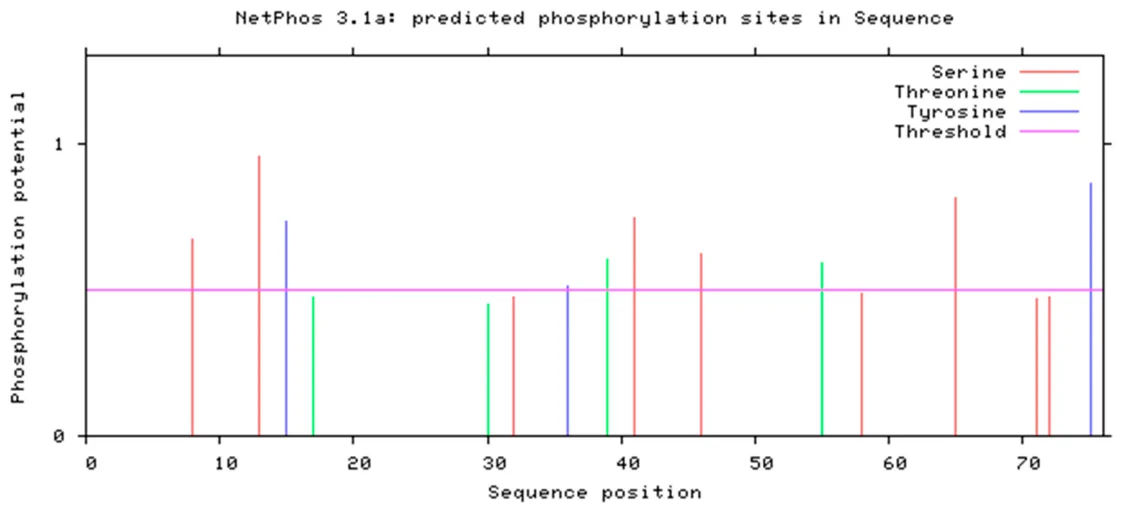
Prediction of phosphorylation sites of ChTp258 protein.

**Figure 18:**
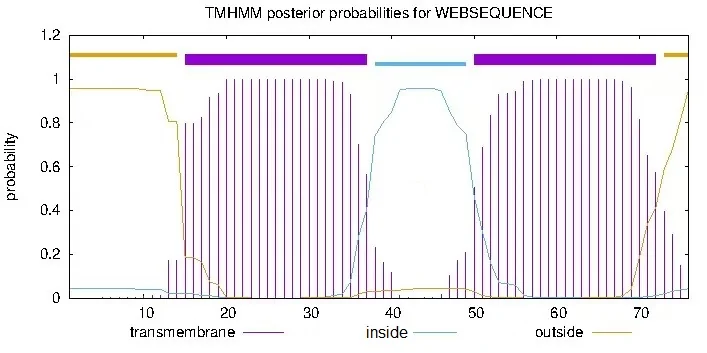
Prediction for transmembrane structures of ChTp258.

**Figure 19:**
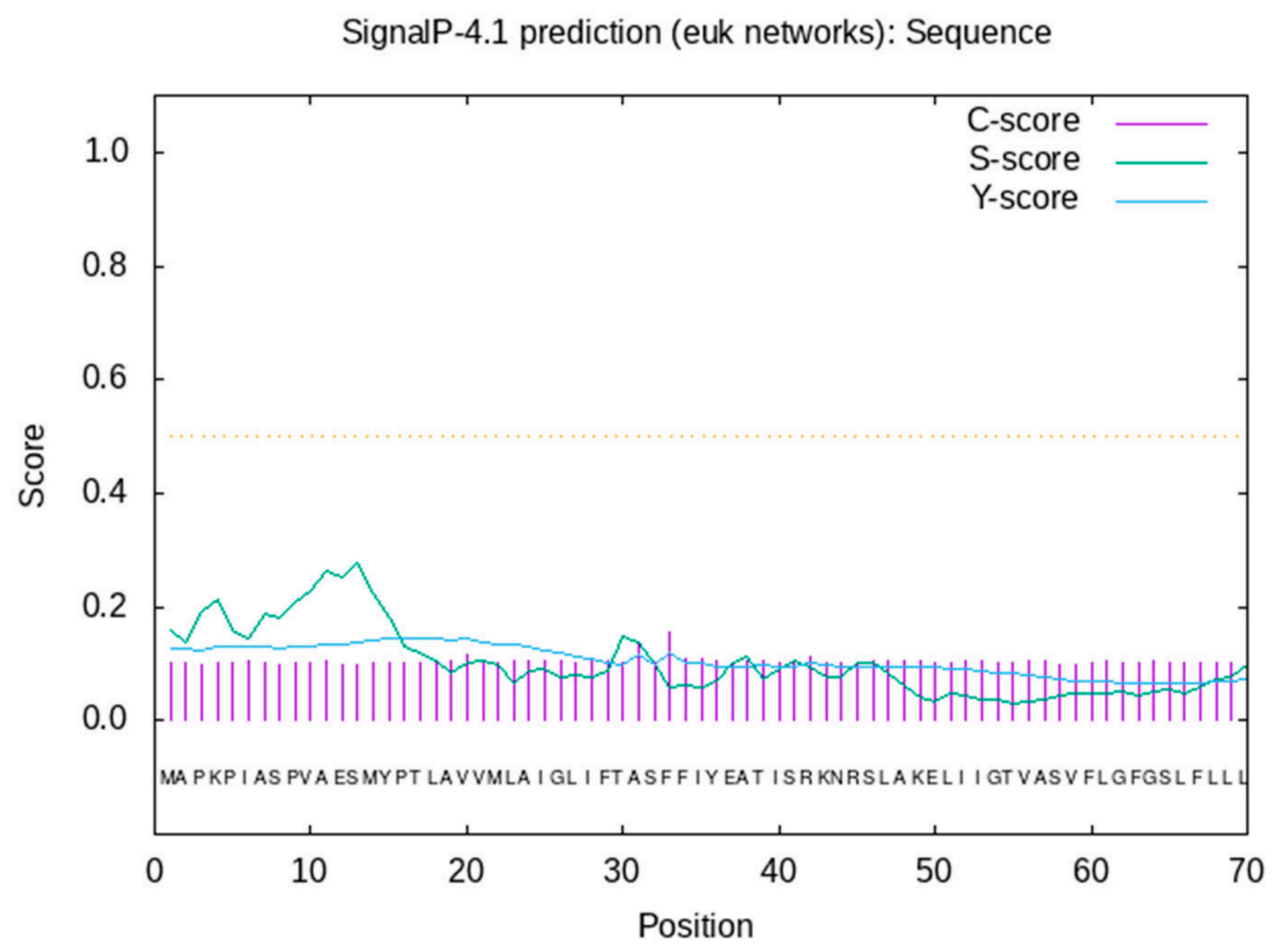
Prediction on signal peptide of ChTp258 protein.

**Figure 20:**
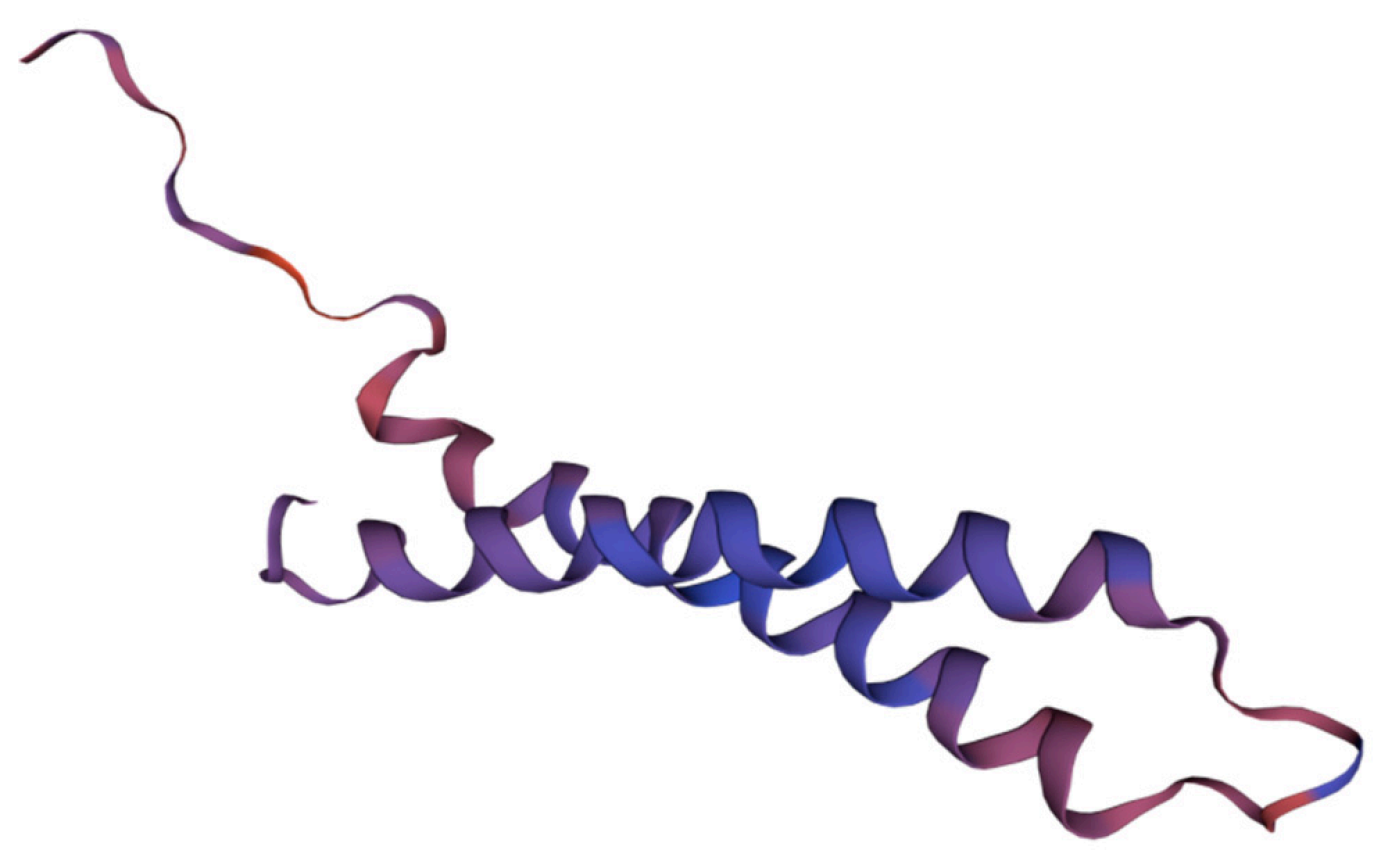
Three-dimensional structure diagram of ChTp258.

Structural features of proteins encoded by ChU40rps27a and ChTp258 were predicted using bioinformatics tools. NetPhos 3.1a analysis identified 14 putative phosphorylation sites in the ChU40rps27a protein, including 4 serine, 8 threonine and 2 tyrosine residues (Figure 13). TMHMM prediction revealed that ChU40rps27a contained no transmembrane domains and was a non-transmembrane protein (Figure 14). SignalP 4.1 analysis indicated that this protein lacked a canonical signal peptide and was not a secreted protein (Figure 15). SWISS-MODEL homology modeling showed that the tertiary structure of ChU40rps27a was mainly composed of α-helices (Figure 16). For the ChTp258 protein, 10 putative phosphorylation sites were predicted, consisting of 5 serine, 2 threonine and 3 tyrosine residues (Figure 17). TMHMM analysis demonstrated that ChTp258 possessed two transmembrane domains and was classified as a transmembrane protein (Figure 18). SignalP 4.1 prediction suggested that ChTp258 had no typical signal peptide and was not a secreted protein (Figure 19). SWISS-MODEL tertiary structure prediction revealed that the three-dimensional structure of ChTp258 was dominated by α-helices with a moderate proportion of random coils (Figure 20).

#### 2.4.3. Multiple Sequence Alignment of ChU40rps27a and ChTp258 Genes from *Cerasus humilis*

**Figure 21:**
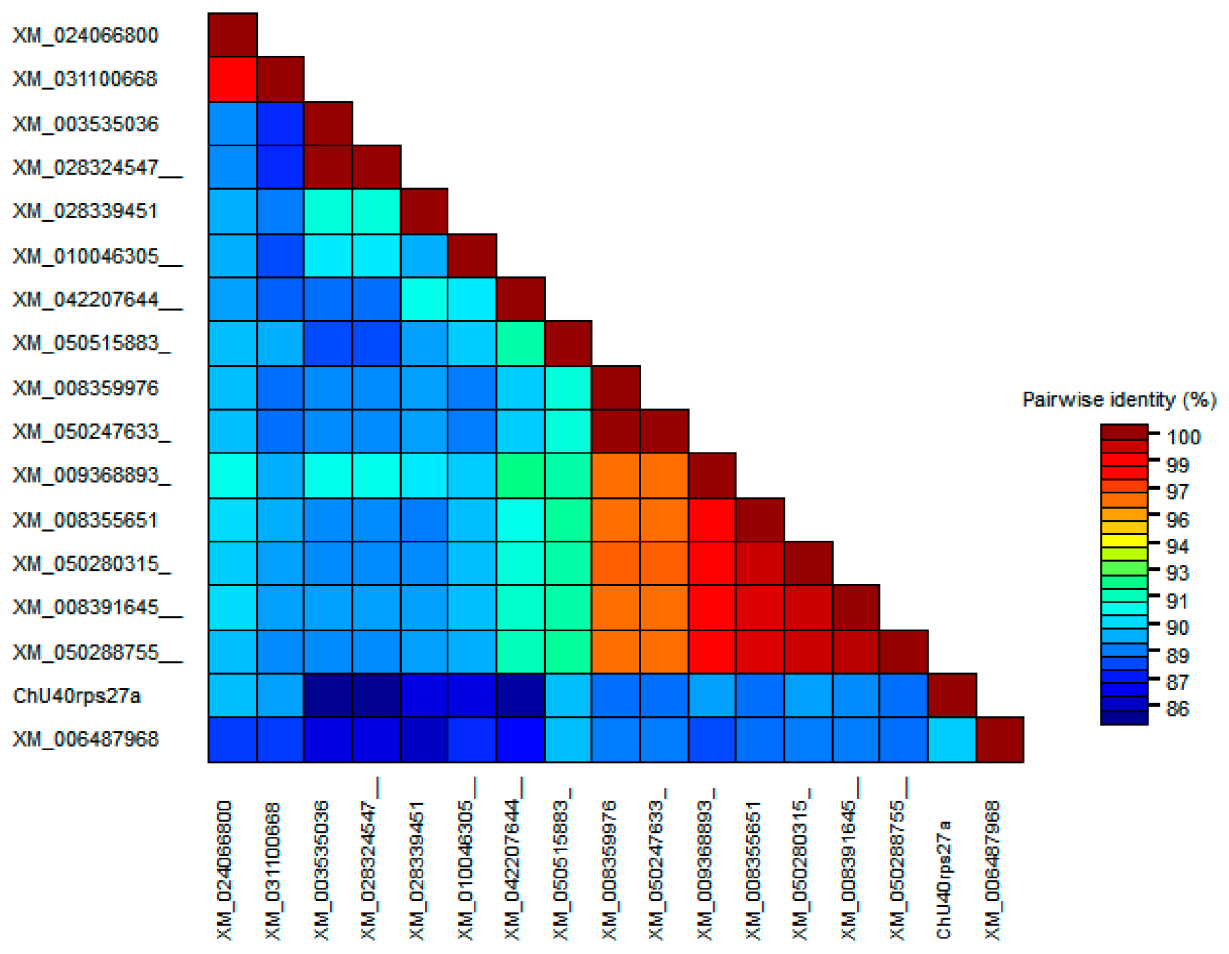
Nucleotide sequence identity analysis of ChU40rps27a with U40rps27a genes from other species.

**Figure 22:**
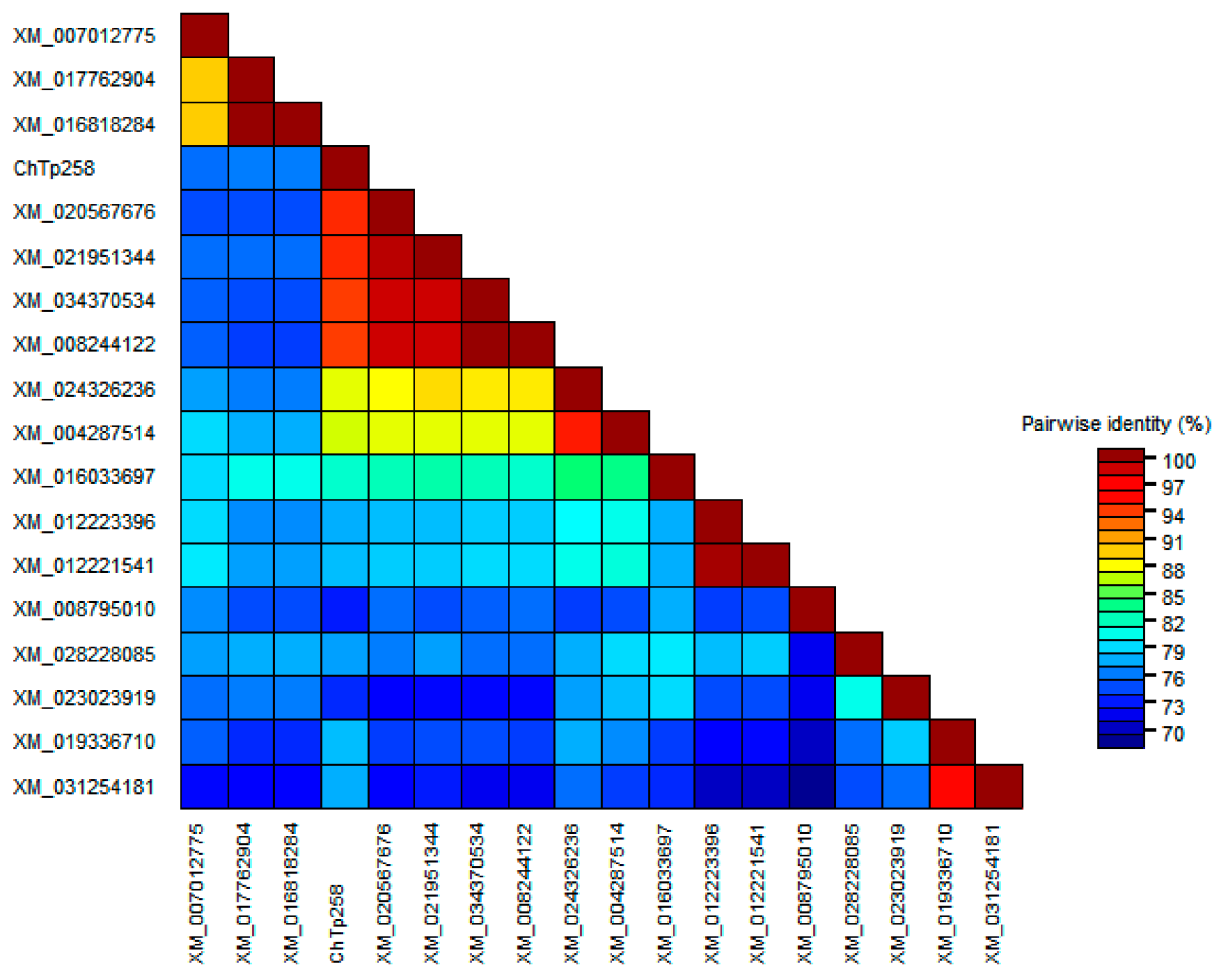
Nucleotide sequence identity analysis of ChTp258 with Tp258 genes from other species.

NCBI BLAST analysis of sequencing results verified that ChU40rps27a and ChTp258 were the target sequences. Homology analysis using SDT v1.0 (Figure 21 and Figure 22) showed that ChU40rps27a shared the highest nucleotide sequence identity of 88.50% with U40rps27a (XM_050288755) from apple (Malus domestica), whereas ChTp258 exhibited the highest nucleotide sequence identity of 94.40% with Tp258 (XM_020567676) from peach (Prunus persica). These results indicated that ChU40rps27a was highly conserved among Rosaceae species, and ChTp258 possessed high sequence conservation in closely related Rosaceae species.

#### 2.4.4. Phylogenetic Analysis of ChU40rps27a and ChTp258 Genes from *Cerasus humilis*

**Figure 23:**
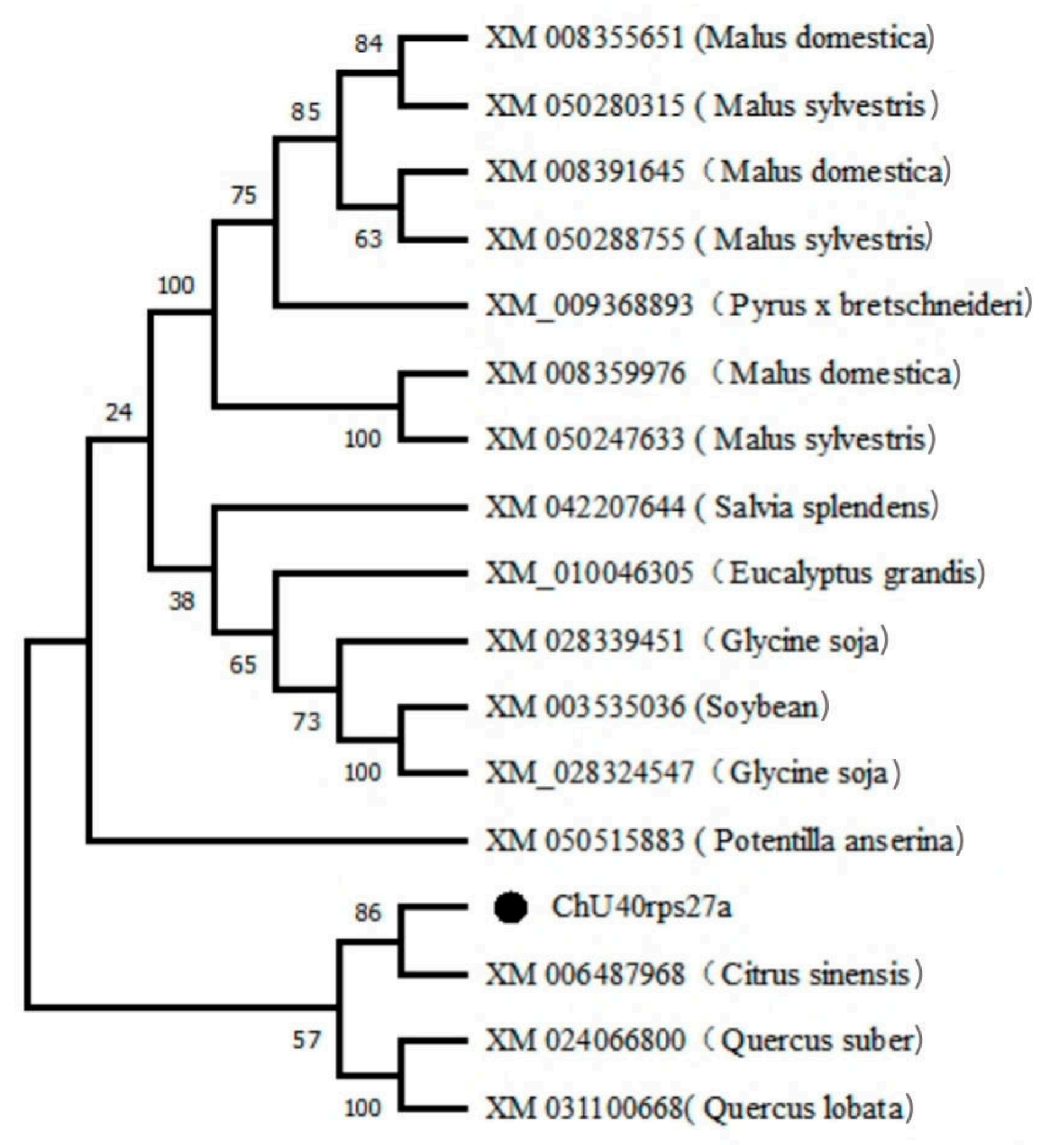
Phylogenetic tree constructed based on U40rps27a amino acid sequences from different species.

**Figure 24:**
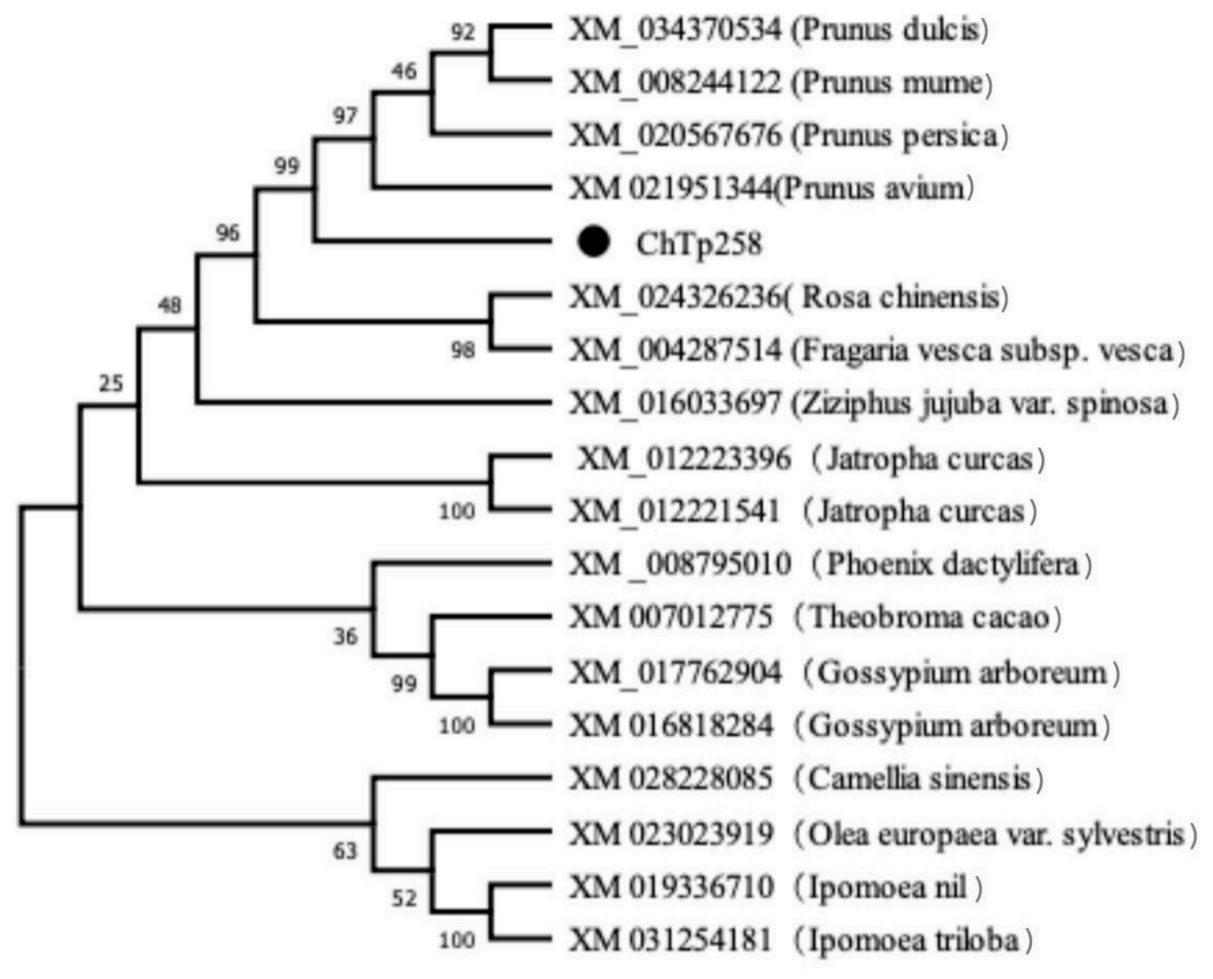
Phylogenetic tree constructed based on Tp258 amino acid sequences from different species.

To investigate the evolutionary relationships of ChU40rps27a and ChTp258, phylogenetic trees were constructed using MEGA 10.0 software based on the amino acid sequences of U40rps27a and Tp258 from different species (Figure 23 and Figure 24). The results revealed that ChU40rps27a had the closest genetic relationship with U40rps27a from sweet orange (*Citrus sinensis*, XM_006487968). ChTp258 was most closely related to species of the genus Prunus within Rosaceae, including peach (*Prunus persica*, XM_020567676), almond (*Prunus dulcis*, XM_034370534), Japanese apricot (*Prunus mume*, XM_008244122) and sweet cherry (*Prunus avium*, XM_021951344), which clustered into a single clade on the phylogenetic tree.

## 3. Discussion

### 3.1. Evaluation of Cerasus humilis cDNA Library

At present, research on yeast cDNA libraries in crops has gradually advanced [15]. The construction and screening of cDNA libraries are among the key approaches for discovering novel genes and investigating gene functions [16], and yeast-based screening represents an important strategy to explore gene regulation and interaction networks [17]. Establishing a high-quality yeast cDNA library is critical for screening interacting proteins. Therefore, when constructing high-quality normalized cDNA libraries using yeast screening technology, library capacity, redundancy and insert fragment size should be taken into consideration, as these factors directly affect protein screening efficiency [18] and prevent screening bias caused by transcriptional level differences [19]. Generally, cDNA library quality is evaluated by its representativeness and the integrity of recombinant sequences. Library capacity reflects representativeness, which indicates the integrity of expressed genetic information (mRNA) in the source tissue [20]. The minimum required clone number for a qualified cDNA library is 1 × 10^6^ cfu [21]. In this study, the constructed *Cerasus humilis* cDNA library exhibited a capacity of 4.52 × 10^7^ cfu/mL with a total clone number of 9.04 × 10^7^ cfu, exceeding the minimum standard and confirming good integrity of the library. PCR identification of 24 randomly selected clones revealed that the average insert fragment size was 1000 bp, demonstrating favorable normalization and suitability for subsequent experimental research.

### 3.2. High-Throughput Screening of Drought-Tolerant Genes via Yeast System

A total of 96 positive yeast clones were obtained through high-throughput screening. After sequence alignment using Seqman and BLAST, empty vectors and redundant sequences were removed, and 44 unique sequences were finally acquired. Reversion verification was performed on these 44 clones using mannitol-simulated drought stress. The negative control strain grew normally on SG-U medium but failed to survive on SG-U medium supplemented with 100 mM mannitol (1:10,000 dilution). In contrast, all 44 screened clones exhibited normal growth on both SG-U and SG-U + 100 mM mannitol (1:10,000 dilution) media. Two candidate genes, ChU40rps27a and ChTp258, were selected from the 44 positive clones and successfully amplified from the genomic sequences of drought-tolerant clones. These results preliminarily confirmed that the 44 positive clones screened by high-throughput technology possess certain drought tolerance under mannitol-simulated drought conditions.

It should be emphasized that our experimental material is total RNA extracted from mixed leaf cell populations (including mesophyll cells, epidermal cells and stomatal cells) after 20 day natural drought treatment. This sampling strategy captures long-term leaf adaptive transcript profiles rather than short-term stress responses (minutes to hours). The yeast heterologous system can only reflect gene mediated osmotic survival in yeast; it cannot fully recapitulate complex multi cell type drought adaptation networks of woody plants. Further plant level genetic validation (e.g., gene silencing or overexpression in *Cerasus humilis*) is required to confirm the in planta biological functions of these candidate genes. Whereas the regulatory mechanisms of these drought-tolerance-related genes remain to be further verified.

### 3.3. Cloning and Bioinformatics Analysis of ChU40rps27a and ChTp258 Genes from Cerasus humilis

In 1975, Goldstein et al. first isolated a small peptide consisting of 76 amino acids from bovine pancreas. This type of small peptide, widely present in eukaryotic cells and organisms, was named ubiquitin (Ub) [22]. The ubiquitin-26S proteasome system specifically and efficiently degrades proteins, with approximately one-third of nascent proteins degraded by this pathway in organisms [23]. Under stress conditions, damaged proteins accumulate in cells, and plant cells selectively degrade these target proteins to maintain normal life activities; the ubiquitin-26S proteasome system is responsible for 80–90% of target protein degradation. Previous studies have demonstrated that TkUBQ6 from Taraxacum kok-saghyz participates in stress resistance and hormone signal transduction [24], and overexpression of ubiquitin genes significantly enhances cold tolerance in transgenic tobacco [25]. In the present study, the ChU40rps27a gene from *Cerasus humilis* was cloned, with an open reading frame (ORF) of 471 bp encoding 157 amino acids. Physicochemical properties, phosphorylation sites, functional domains, transmembrane structures and tertiary structure were predicted. Nucleotide sequence identity analysis revealed the highest similarity (88.50%) with 40S ribosomal protein from apple (*Malus domestica*, XM_050288755). Phylogenetic analysis indicated that ChU40rps27a was most closely evolutionarily related to U40rps27a from sweet orange (*Citrus sinensis*, XM_006487968). At present, the regulatory mechanism of this gene in stress response of *Cerasus humilis* remains unclear, which will be a key direction for further research.

As signal transducers between cells and the external environment, transmembrane proteins are involved in multiple physiological processes including vitamin transport, lipid metabolism, pigment synthesis, carbon metabolism and mitochondrial ATP synthesis, and play vital roles in plant stress tolerance. It has been reported that tobacco NtSyp121 is regulated by ABA, drought and salt stresses; disruption of its functional domains inhibits vesicle trafficking and blocks ABA-related ion channels. With dual functions in membrane fusion and ion channel interaction, it further regulates stomatal movement [25]. The cloned ChTp258 gene of *Cerasus humilis* had an ORF of 231 bp encoding 77 amino acids. Predictions of physicochemical properties, phosphorylation sites, functional domains, transmembrane structures and tertiary structure were performed. Nucleotide sequence alignment showed the highest identity (94.40%) with Tp258 from peach (*Prunus persica*, XM_020567676). Phylogenetic analysis demonstrated that ChTp258 clustered into a single clade with orthologs from peach (XM_020567676), almond (*Prunus dulcis*, XM_034370534), Japanese apricot (*Prunus mume*, XM_008244122) and sweet cherry (*Prunus avium*, XM_021951344). The molecular regulatory mechanism of ChTp258 underlying drought tolerance in Cerasus humilis is still unclear and requires further investigation.

Plant drought tolerance is a complex process involving multiple molecular regulatory pathways and metabolic processes. The two genes ChU40rps27a and ChTp258 identified in this study provide fundamental evidence for further exploring drought-tolerance mechanisms and molecular breeding of resistant varieties in *Cerasus humilis*.

## 4. Materials and Methods

### 4.1. Construction of Cerasus humilis cDNA Library

Two-year-old wild *Cerasus humilis* plants were cultivated in a greenhouse located at the Inner Mongolia Education Experimental Base in Hohhot. The photoperiod was 14 h light/10 h dark, day temperature was 24–28 °C, night temperature was 16–20 °C, relative humidity was 30–45%. For natural drought treatment, irrigation was completely withheld for 20 consecutive days and leaf samples were collected and snap-frozen in liquid nitrogen and stored at −80 °C for subsequent use. Escherichia coli TOP10 strain was cultured in liquid LB medium at 37 °C and preserved at −80 °C with 20% glycerol.

Total RNA was extracted from mixed leaf-cell populations including mesophyll, epidermal and stomatal guard cells via the Trizol method. Clear RNA bands were observed, and the OD_260_/_280_ ratio ranged from 1.8 to 2.2, indicating qualified RNA quality. Messenger RNA was enriched and purified using Oligo (dT) magnetic beads, which was then reversely transcribed into single-stranded cDNA. Double-stranded cDNA was amplified by three-frame primer PCR and high-purity products were recovered with DNA purification magnetic beads.

The double-stranded cDNA was subjected to denaturation, hybridization and DSN normalization. Small fragments were removed by centrifugal chromatography column after PCR amplification to obtain normalized cDNA [26]. The purified double-stranded cDNA was homologously recombined with linearized pYES2 vector, and the recombinant products were electrotransformed into competent E. coli TOP10 cells. After resuscitation, bacterial cells were spread on LB-Amp plates and incubated overnight at 37 °C [27]. Library identification results showed that the titer of the constructed cDNA library was 4.52 × 10^7^ cfu/mL, the average insert fragment length was 1000 bp, and the recombination rate reached 96%. Library plasmids were extracted using the Tiangen large-scale plasmid extraction kit (Tiangen Biotech (Beijing) Co., Ltd., Beijing, China) and stored at −20 °C.

### 4.2. High-Throughput Screening of Drought-Tolerant Genes in Yeast

Using pYES2 as the expression vector, YPDA was adopted as the complete yeast medium, while SD-U and SG-U served as uracil-deficient selective media. 10 × TE and 10 × LiAc buffers were prepared and autoclaved for standby use [28]. Saccharomyces cerevisiae strain BY4741 was activated and cultivated until the OD_600_ value reached 0.6. The harvested cells were washed and resuspended with sterile water and lithium acetate solution [29]. Mixtures of PEG, LiAc, ssDNA and plasmids were added for incubation. After heat shock at 42 °C and recovery at 30 °C, the cell suspension was spread onto selective plates and cultured at 30 °C for 4 days. Positive clones were verified by PCR assay. The constructed *Cerasus humilis* cDNA library plasmids were transformed into competent BY4741 yeast cells. The cells were cultured on SG-U plates at 30 °C for 3 to 7 days. Colonies were collected to prepare working bacterial liquid and preserved at low temperature. The transformation quality of the library was evaluated through gradient dilution counting and colony PCR detection [30]. Positive yeast strains were activated and induced. Gradient diluted bacterial solutions were spotted onto plates supplemented with different concentrations of mannitol for drought stress screening, and 100 mM mannitol was confirmed as the optimal stress concentration. Library plates were spread and 96 single colonies were picked for sequencing and alignment. Redundant sequences were eliminated, and functional verification of candidate drought-tolerant genes was accomplished via yeast complementation assay [31].

### 4.3. Cloning and Bioinformatics Analysis of Drought Stress-Related Genes

Total RNA was extracted using Spectrum^TM^ Plant Total RNA Extraction Kit (Merck Life Science, Shanghai, China). After passing quality inspection, the first-strand cDNA was synthesized with ABScript III Reverse Transcription Kit (ABclonal Technology Co., Ltd., Wuhan, China) [32]. Two candidate drought-tolerant genes ChU40rps27a and ChTp258 were screened based on yeast drought tolerance screening results. Specific primers were designed according to conserved gene sequences (Table 1). PCR amplification was performed using cDNA as template. The amplified products were recovered from agarose gel, subjected to TA cloning and transformed into competent cells, followed by screening and sequencing verification of positive clones. NCBI Blast was used for gene homologous sequence alignment. Online tools including ExPASy (accessed in March 2023), ProtScale (accessed in March 2023), NetPhos 3.0, SignalP 6.0 and SWISS-MODEL (accessed in March 2023) were applied to predict physicochemical properties, modification sites and spatial structure of encoded proteins. Sequence homology analysis and phylogenetic tree construction were conducted via SDT and MEGA 10.0 software.

## 5. Conclusions

A normalized yeast cDNA library was constructed from drought-stressed leaf tissues of *Cerasus humilis*. The library capacity was 4.52 × 10^7^ cfu/mL with a total clone number of 9.04 × 10^7^ cfu. Twenty-four randomly selected clones were subjected to colony PCR, revealing an average insert fragment size of 1000 bp and a recombination rate of 96%. High-throughput yeast screening was used to identify drought-tolerant genes, yielding 96 positive yeast clones. After PCR amplification, sequence alignment with SeqMan and BLAST, and removal of empty vectors and redundant sequences, 44 unique sequences were obtained. Reversion verification using mannitol-simulated drought stress showed that all 44 clones grew normally on both SG-U medium and SG-U medium supplemented with 100 mM mannitol (1:10,000 dilution).

Further analysis was performed on the 44 candidate genes and proteins responsive to drought stress in *Cerasus humilis* leaves. Two drought-tolerant candidate genes, ChU40rps27a and ChTp258, were successfully amplified. Their open reading frames were 471 bp and 231 bp, encoding 157 and 77 amino acids, respectively. Nucleotide sequence identity and phylogenetic analyses showed that ChU40rps27a shared the highest identity of 88.50% with U40rps27a from apple (*Malus domestica*, XM_050288755), and was most closely evolutionarily related to U40rps27a from sweet orange (*Citrus sinensis*, XM_006487968). ChTp258 exhibited the highest nucleotide identity of 94.40% with Tp258 from peach (*Prunus persica*, XM_020567676), and clustered into one clade with orthologs from peach (XM_020567676), almond (*Prunus dulcis*, XM_034370534), Japanese apricot (*Prunus mume*, XM_008244122) and sweet cherry (*Prunus avium*, XM_021951344).

In summary, these findings provide a theoretical basis for further elucidating the drought-resistance mechanism of wild *Cerasus humilis*. Several key drought-responsive genes and proteins were identified, which is of great significance for theoretical analysis of drought tolerance and molecular breeding for drought resistance in wild *Cerasus humilis*.

## Figures and Tables

**Table 1:** Primers used to clone the sequence of ChU40rps27a, ChTp258 genome.

Primers	Sequences (5′ → 3′)	Tm (°C)	Size (bp)	Target Fragment
ChU40rps27aF	ATGCAGATCTTCGTGAAAACCC	54	22	471
ChU40rps27aR	CTAATCACCGCCAGCCTTCT	54	20	
ChTp258F	ATGGCCCAATCAGCGAAGC	52	19	231
ChTp258R	TCAAACGTAAACACCACAGGA	50	24	

Note: The primer pairs in Table 1 were designed to fully cover the complete open reading frame (ORF) of each target gene based on sequencing reads obtained from positive yeast clones. These ORF specific primers guarantee amplification of full length coding sequences for subsequent cloning and sequence verification.

## Data Availability

The data presented in this study are available on request from the corresponding author due to ethical restrictions on participant privacy.
